# A global review of past land use, climate, and active vs. passive restoration effects on forest recovery

**DOI:** 10.1371/journal.pone.0171368

**Published:** 2017-02-03

**Authors:** Paula Meli, Karen D. Holl, José María Rey Benayas, Holly P. Jones, Peter C. Jones, Daniel Montoya, David Moreno Mateos

**Affiliations:** 1 Natura y Ecosistemas Mexicanos A.C., Mexico DF, Mexico; 2 Fundación Internacional para la Restauración de Ecosistemas, Madrid, Spain; 3 Environmental Studies Department, University of California Santa Cruz, Santa Cruz, California, United States of America; 4 Departamento de Ciencias de la Vida, Universidad de Alcalá, Alcalá de Henares, Spain; 5 Department of Biological Sciences and Institute for the Study of the Environment, Sustainability, and Energy, Northern Illinois University, DeKalb, Illinois, United States of America; 6 Department of Biological Sciences, Northern Illinois University, DeKalb, Illinois, United States of America; 7 Centre for Biodiversity Theory and Modeling, Station D’Ecologie Experimentale du CNRS, Moulis, France; 8 Centre INRA de Dijon, Dijon Cedex, France; 9 School of Biological Sciences, University of Bristol, Bristol, United Kingdom; 10 Basque Center for Climate Change – BC3, Bilbao, Spain; 11 IKERBASQUE, Basque Foundation for Science, Bilbao, Spain; Kerala Forest Research Institute, INDIA

## Abstract

Global forest restoration targets have been set, yet policy makers and land managers lack guiding principles on how to invest limited resources to achieve them. We conducted a meta-analysis of 166 studies in naturally regenerating and actively restored forests worldwide to answer: (1) To what extent do floral and faunal abundance and diversity and biogeochemical functions recover? (2) Does recovery vary as a function of past land use, time since restoration, forest region, or precipitation? (3) Does active restoration result in more complete or faster recovery than passive restoration? Overall, forests showed a high level of recovery, but the time to recovery depended on the metric type measured, past land use, and region. Abundance recovered quickly and completely, whereas diversity recovered slower in tropical than in temperate forests. Biogeochemical functions recovered more slowly after agriculture than after logging or mining. Formerly logged sites were mostly passively restored and generally recovered quickly. Mined sites were nearly always actively restored using a combination of planting and either soil amendments or recontouring topography, which resulted in rapid recovery of the metrics evaluated. Actively restoring former agricultural land, primarily by planting trees, did not result in consistently faster or more complete recovery than passively restored sites. Our results suggest that simply ending the land use is sufficient for forests to recover in many cases, but more studies are needed that directly compare the value added of active versus passive restoration strategies in the same system. Investments in active restoration should be evaluated relative to the past land use, the natural resilience of the system, and the specific objectives of each project.

## Introduction

The current awareness of society’s dependence on forests underpins recent international initiatives to halt deforestation and increase restoration of the more than two billion hectares of degraded forests globally [1, 2]. Most notable of these are the 2011 Bonn Challenge and the 2014 New York Declaration on Forests, which aim to restore 150 million hectares of forest worldwide by 2020 and 350 million hectares by 2030, respectively [3]. These goals align with the Aichi target of restoring at least 15% of degraded ecosystems by 2020 [4]. Restoration decision makers need guiding principles regarding how to invest limited resources for large scale forest restoration to achieve these goals [5].

**Fig 1:**
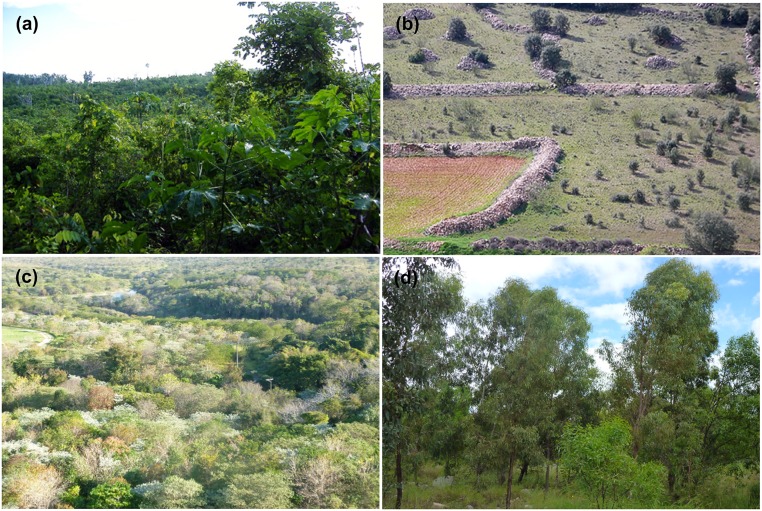
Examples of passive and active forest recovery worldwide. (a) Three-year-old natural regeneration in a dry forest site in the Yucatan Peninsula, Mexico that was previously used for shifting agriculture; (b) a ~12-year-old natural regeneration in former agricultural land in central Spain; (c) a 300-ha, 6-year-old site in the Atlantic forest of Brazil restored by planting >60 species of trees; (d) a ~7-year-old former coal mine in Queensland Australia restored to a eucalypt woodland by recontouring topography, seeding and planting native species, and fertilizing. Photo credits: (a)–Martha Bonilla-Moheno, (b)—José M. Rey Benayas; (c)–Karen Holl, and (d)–Carl Grant.

A comprehensive global analysis of forest recovery is timely given the substantial resources being invested in forest restoration globally and regionally. Previous analyses have mostly focused on specific forest types (e.g., tropical), restoration approaches (e.g., secondary succession), and/or restoration outcomes (e.g. biodiversity or carbon accumulation) [6–9]. Predicting the rate and degree of forest recovery is challenging as both can be affected by a number of factors, such as past land use type, forest region, landscape context, and restoration approach [10–12]. In some cases, forests recover quickly when the impeding past land use (e.g., agriculture, logging) ceases and natural succession can proceed [13, 14] (hereafter, passive restoration, Table 1; Fig 1a and 1b). Meanwhile, in areas with extensive deforestation, a combination of limited seed dispersal, aggressive exotic vegetation, microclimatic extremes, and/or soil degradation can result in slow or no recovery [15]. Thus people often intervene in various ways to accelerate recovery, such as planting trees, amending soil, and recontouring topography (hereafter, active restoration; Table 1; Fig 1c and 1d).

**Table 1:** Glossary.

*Active restoration*: a range of human interventions in an effort to accelerate and influence the successional trajectory of recovery [10].
*Degradation level*: the degree to which the degraded conditions differ from the reference level.
*Degraded conditions*: measurements taken soon after the prior land use ceased.
*Ecological restoration*: the process of assisting the recovery of an ecosystem that has been degraded, damaged, or destroyed [25].
*Passive restoration*: ending the prior anthropogenic land-use type to allow the forest for natural or unassisted recovery [10].
*Recovery completeness*: the degree to which a metric type (i.e., abundance, diversity, and biogeochemical functions) measured in the restored site attains the reference level.
Reference conditions: the prior land use conditions or data from intact or minimally disturbed sites, as defined by the primary studies used in this meta-analysis.

Past studies have reported that the rate of forest recovery may vary substantially depending on the type of variable measured (e.g., biodiversity, biogeochemical cycling) [9, 12, 16]. Planting trees can speed up the recovery of forests (e.g., biodiversity — [17], carbon storage — [18]) and their economic benefits to societies [19]. In other cases, active forest restoration can negatively affect natural regeneration [20], result in less diverse forests [21, 22], produce ‘disservices’ such as reducing soil moisture [23], or may not be cost-effective [24].

Here, we compiled a large database of forest restoration studies globally to make robust conclusions about factors that affect forest recovery and, specifically, evaluate the value added of active forest restoration efforts. We evaluated the relative importance of several context-dependent factors underpinning outcomes of forest restoration. We analyzed restoration of floral and faunal abundance and diversity, and biogeochemical functions related to carbon, nitrogen and phosphorus compared to reference conditions (Table 1) and asked three main questions: (1) To what degree do floral and faunal abundance and diversity, and biogeochemical functions recover? (2) Does recovery vary as a function of past land use, time since restoration/recovery began, forest region, or precipitation? (3) Does active restoration result in more complete or faster recovery than passive restoration? The answers are important for prioritizing restoration actions.

## Materials and methods

### Literature review

We compiled data related to forest recovery, either passive or active, mainly from a systematic literature search on ISI Web of Science (65% of total studies in this meta-analysis, see S1 File for a detailed description of database compilation), as well as 26% from the database in [26], 8% from [16], and 1% from [27]. Given our objectives and data availability, we focused on those papers evaluating forest recovery after the three most common previous land-use types: agriculture, logging, and mining. We compared restored conditions with reference conditions (as defined in the primary study) to assess recovery completeness and with degraded conditions to quantify the extent of initial degradation levels (Table 1). We included a total of 1,804 response variables from 166 studies.

We compiled metrics of restoration outcomes and several potential predictor variables that could influence recovery completeness (see S1 File for full variable list and descriptions). Metric types were abundance, diversity (species, genus or family richness, and evenness) or biogeochemical function (nutrient concentrations or fluxes). Potential predictor variables were (1) specific life form (vegetation—both woody and non-woody, invertebrate, or vertebrate) or biogeochemical function (above-ground carbon, below-ground carbon, nitrogen, or phosphorus), (2) forest region (tropical = <23.5° latitude or temperate = >23.5°), (3) precipitation (wet or dry, with mean annual precipitation >2000 mm or ≤ 2000 mm per year, respectively), (4) past land-use type (agriculture, logging, or mining), (5) restoration approach (passive or active), and (6) time since restoration started. Most of the studies reported multiple response measures (e.g., abundance data for different taxonomic groups).

### Data analysis

To answer Question 1 we used transformed response ratios (RR) as the effect size to estimate recovery completeness [28], which is commonly used in ecological meta-analyses [29]. We computed RR as ln(X_res_+ 0.001)/(X_ref_+ 0.001), where X_res_ is the variable measured in the restored forest (current condition) and X_ref_ is the reference measure selected by the author(s) of the primary study, either from a nearby reference forest or from data prior to human disturbance [16]. The response ratios are log-transformed proportional differences between two conditions, which we report throughout as percentage values (by back-log transforming the response ratios) to enhance clarity. Full recovery of a particular metric is indicated when the confidence intervals (CI) for recovery completeness overlap 100%. We also compared the magnitude of degradation levels prior to restoration after different past land-use types and in active vs. passively restored sites to help interpret our recovery results as ln (X_deg_+0.001)/(X_ref_+0.001), where X_deg_ is the value of the response variable in the degraded forest, and values less than 0 indicate degraded conditions.

Response ratios tend toward a value of zero as X_res_ increases to a value approaching X_ref_. Whereas increases in most response variables indicate improvement, increases in others indicate degradation. For example, an increase in the abundance or richness of non-native species implies reductions in biodiversity. Hence, we reversed the sign of the response ratios for those metrics that were expected to decrease as a result of ecological restoration. Response ratios close to zero indicate restored forests with similar values to reference forests, and negative response ratio values indicate restored forest values that are lower than the reference forest.

Adding 0.001 to both the numerator and denominator of the response ratio avoids zero values and linearizes the response variable. A scatter-plot showed that this transformation did not affect our final results (S1a Fig). A plot of standardized recovery completeness effect sizes against the normal quantiles [30] showed a slight deviation from a normal distribution but the sample size was large (n = 1,804) thus minimizing the effect of this deviation (S1b Fig). In addition, we reran the analyses removing the most extreme outliers and the results did not change, so we included all data.

Meta-analyses are often weighted by the inverse variance of the response ratio in each study [28]. However, as is commonplace with ecological meta-analyses [9, 16], we were unable to conduct weighted analyses because mean, standard deviation, and sample size information for each response variable were available for only 35% of total cases. Thus, a weighted analysis would have excluded the majority of our data.

To answer Question 2, we used general linear mixed models and the Akaike Information Criterion (AIC), an information-theoretic approach based on likelihood measures, to compare several models after model averaging [31]. We constructed a set of candidate models that included several predictor variables as both additive and interactive effects among them as fixed effects. To account for non-independence when including multiple response variables from the same primary study, all models included a ‘study’ variable as a random effect [32]. We used the same modeling approach to analyze initial degradation levels (S2a and S3a Tables).

For the full data set we included forest region, precipitation, past land-use type, time since restoration started, and life form or biogeochemical function; we included two-way interaction terms for which we had a sufficient sample size for potential combinations of predictor variables (i.e., at least three studies and 15 data points). When we compared models for recovery of all metric types simultaneously, the model that best explained the variation in the data included several interactions between metric type and other predictor variables (S2b and S3b Tables). Thus, we compared models for the different metric types separately to determine which factors best predicted recovery of each one. In comparing active vs. passive restoration in former agricultural sites (see below), we included forest region and time in these models, given that these factors were most important in explaining the full data set; however, we did not have a sufficient sample size to include other predictor variables in the models. Time since restoration actions started ranged from six months to 300 years, but only 7% of the studies reported on sites >100-yr-old. So, we ran all these analyses excluding >100-yr-old studies and results did not change.

Ecological questions are fundamentally complex and may require several variables to be considered simultaneously for inference. As a consequence, the various candidate models involving different sets of parameters can be considered as competing hypotheses [33, 34]. We considered any models with a ΔAIC ≤ 2 to have comparable support [35] and used model averaging to compare models. In this approach models with the lowest AIC, and thus highest Akaike weight (i.e., the relative likelihood of the model being the best), are considered to best fit these data. The model weight (w_i_) can be interpreted as equivalent to the probability that a model is the best fitting data among all candidate models. The ‘relative importance’ or ‘predictor weight’ (Ʃw) of each variable under consideration can be interpreted as equivalent to the probability that a predictor is a component of the selected model [36]. The relative importance of each variable is calculated by adding the Akaike weights of all models that included that variable [37].

**Fig 2:**
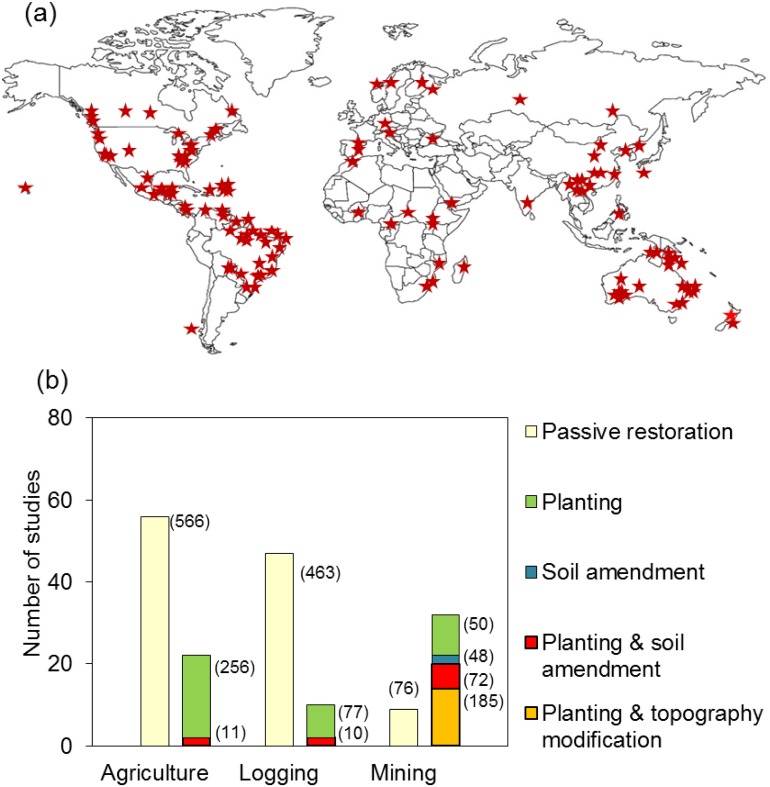
**(a) Geographic distribution of studies. (b) Number of studies using passive or different active restoration actions** as a function of the main past land-use types. Numbers of response variables in each category are indicated in parentheses.

We used a similar modeling approach to compare active and passive restoration approaches (Question 3). There were few studies of active restoration in previously logged forests and in >40-yr-old agricultural sites, and of passive recovery on mined sites (Fig 2b). Thus we focused our comparison of active vs. passive restoration on ≤40-yr-old agricultural sites, given that these were the only conditions where sample sizes were sufficient to make robust comparisons. We conducted an additional analysis of the few studies reporting data for both passive and active restoration approaches within the same site but did not have a sufficient sample size to analyze by metric type.

All analyses were conducted in R v.3.0.2 using the ‘nlme’ package for linear mixed effects models [38], ‘MuMIn’ package for model averaging [39], and the ‘ggplot2’ package for predictions [40]. We visualize recovery across time of linear model predictions only for the highest weighted variables.

## Results and discussion

### Overview of forest recovery database

The 166 studies were distributed across 132 locations in 41 countries (Fig 2a). Both temperate (56% of studies) and tropical forest (44%) as well as wet (55%) and dry (45%) forests were evenly represented (S1 Table). Most studies (44%) were from former agricultural sites, followed by logged (34%) and mined sites (22%). A higher proportion of studies quantified recovery following passive restoration (67%) than recovery of actively restored sites (40%; Fig 2b); only 11 studies (7%) reported data for both passive and active restoration approaches within the same site. Nearly all actively restored sites included tree planting as the main restoration action, except for a few mined sites (Fig 2b). A higher proportion of studies reporting abundance and diversity response variables measured vegetation (62% abundance, 50% diversity) than invertebrates (30%, 39%) or vertebrates (15%, 22%). Of the studies reporting biogeochemical function values, 65% measured below-ground carbon, 49% above-ground carbon, 42% nitrogen, and 30% phosphorus. Time since restoration actions started varied between six months and 300 years (median time 18.5 years); 93% of the studies were with <100 years since restoration started. The model including past land-use type had a similar AIC value as the null model (S2a Table), suggesting that degradation levels in agricultural, mined and logged sites did not differ strongly. However, we observed a trend toward slightly more degraded conditions in agricultural than logged sites (S2a Fig).

### Effect of past land use, forest region, precipitation, and time since restoration on recovery

Across all studies (regardless of time since restoration started), abundance recovered to levels higher than reference sites (146.1%, CI: 124.4, 171.5), diversity barely reached the reference levels (83.0%, CI: 68.7, 100.3), and biogeochemical functions were not fully recovered (81.3%, CI: 69.5, 95.0). A model including metric type (i.e., abundance, diversity, and biogeochemical functions), time since restoration started, and their interaction best explained overall differences in recovery completeness (S2b and S3b Tables), and thus we ran separate models for each metric type.

**Fig 3:**
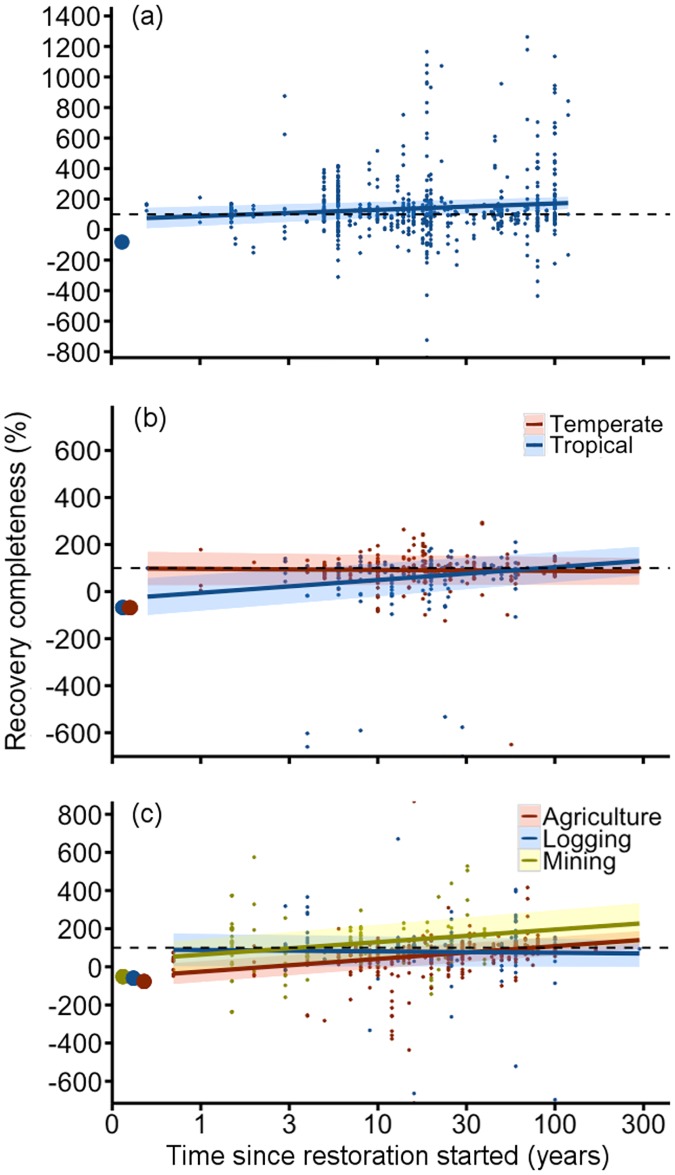
Recovery completeness over time in all forest restoration studies. (a) Abundance, (b) diversity and (c) biogeochemical functions separated by the explanatory factors that explained the most variation in recovery completeness according to model averaging. Colored lines illustrate means and shaded areas indicate 95% confidence intervals of predicted recovery values based on models. Confidence intervals overlapping the black dashed lines indicate full recovery. Circles at time 0 indicate the mean degraded value before restoration started calculated from raw data. Small dots represent raw data. Note log scale for time since restoration on x-axis. See ‘Materials and methods’ for modeling details.

Although no single model for abundance recovery had higher support than the null model, time since restoration started was the predictor variable with the highest weighting (S2c and S3c Tables). Abundance was much lower in the degraded state, but it recovered within a few years and the values observed in older restored sites were often higher than those observed in the reference forests (Fig 3a).

**Fig 4:**
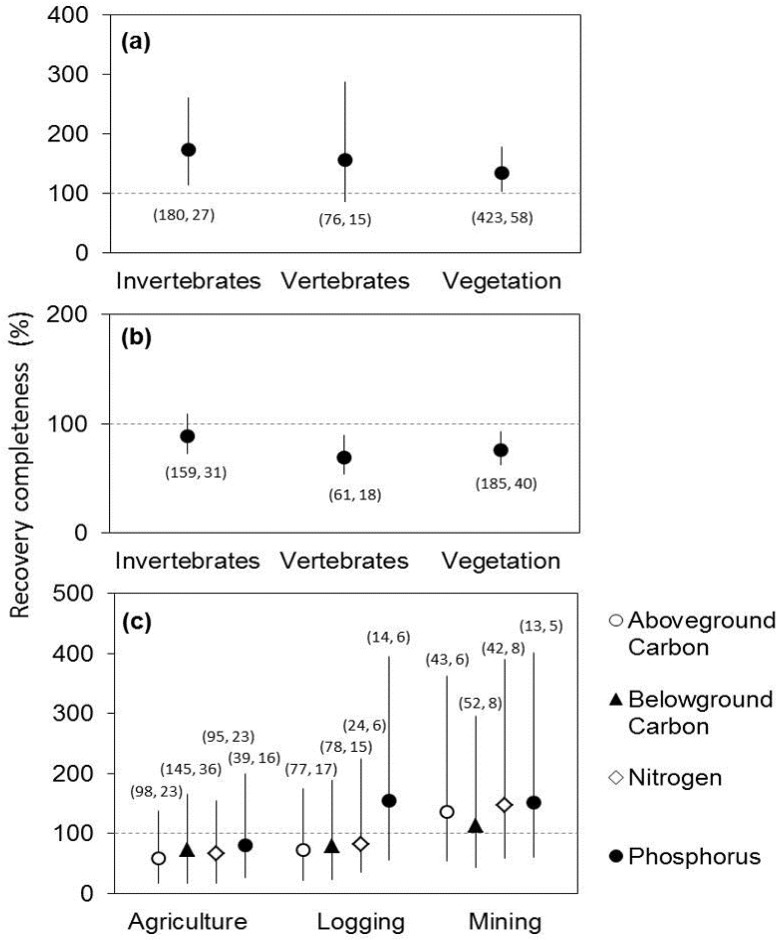
Recovery completeness of different life forms and biogeochemical functions after the three past land-use types. (a) Abundance and (b) diversity of different life forms, and (c) biogeochemical functions. Number of response variables and studies in each category are indicated in parentheses.

Time since restoration started and forest region were the strongest predictors of diversity recovery (S2d and S3d Tables). Diversity recovered in temperate forests regardless of time since restoration started (Fig 3b). In the tropics, however, diversity was lower in younger restored forests than in reference forests and increased with time since restoration started; mean values reached reference levels after ~30 years (Fig 3b). Invertebrates, vertebrates, and plants showed similar trends in recovery of abundance and diversity (Fig 4a and 4b; S2c, S2d and S3c, S3d Tables).

For recovery of biogeochemical functions (above-ground C, below-ground C, N, and P), past land-use type and time since restoration started were the factors with highest model weighting (S2e and S3e Tables). On average, biogeochemical functions in restored logged and mined sites were similar to reference values regardless of the time since restoration started (Fig 3c); this counterintuitive result is likely due to the high variance across sites of similar ages. The recovery of biogeochemical functions was lower in recently restored agricultural sites and increased with time to approach full recovery in ~30-year old sites. Recovery completeness did not differ among specific biogeochemical functions (Fig 4c; S2e and S3e Tables). Precipitation (wet or dry) had low weightings in both the overall and all individual metric type models (S2b–S2e and S3b–S3e Tables).

### Comparison with previous ecosystem recovery studies

Our results showed that, for the metric types we analyzed, recovery happened relatively quickly and, on average, they reached reference values within the first few decades since restoration started. These findings are consistent with an earlier global meta-analysis of ecosystem recovery that showed forest recovery within 42 years [26]. Other meta-analyses in aquatic and terrestrial ecosystems have reported a lower degree of recovery and a much longer time lag (e.g., on the order of over a century) in recovery [7, 9, 41]. One explanation for the higher and faster degree of recovery in our study may be that we focused on abundance, diversity, and biogeochemical functions, whereas other metrics such as community composition, phylogenetic diversity, species interaction networks, and functions derived from species interactions (e.g., propagule dispersal) may recover more slowly [9, 42]. For example, community similarity to reference forest is typically a slow metric to recover [9]; since recovering forests are typically dominated by a suite of pioneer and generalist species whereas rarer species are absent, it is critical to protect existing forests to conserve dispersal-limited, primary forest specialists which recolonize restored sites slowly, if at all [43, 44].

Our results concur with previous studies showing that degree of recovery varies strongly depending on the past land use and the metric type measured [8, 9, 12, 26, 41]. For instance, abundance sometimes reached higher values than reference systems in older sites. This result is consistent with many studies of forest chronosequences showing that tree density recovers quickly within the first few years of succession and then declines as the forest matures (e.g., [14, 45]). It also raises the common assumption of linearity of ecosystem recovery between a starting point and an end point, typically relative to reference systems [16, 46], which is not necessarily true [47]. Linear trends are straightforward to assess for restoration goals focusing on ecosystem services such as carbon sequestration and soil protection, but not for other recovery indicators such as abundance, diversity and species composition. This variation highlights the need to evaluate ecosystem recovery using multiple metrics over time to be able to better characterize the shape of recovery trajectories, but few studies report data from sufficient time points to do so.

Measures of diversity recovered completely in younger sites in temperate as compared to tropical systems. A larger species pool in the tropics than in the temperate zone is one possible explanation of this difference. A high degree of dispersal limitation in tropical forests [15] also could partially explain the generally slower recovery of diversity in the tropics.

Past land-use type explained the most variation in the recovery of biogeochemical functions; former agricultural land recovered more slowly, but variance was high, consistent with [26] and [48]. This result suggests that the impacts caused by agriculture (e.g., soil eutrophication, depletion of organic matter) have strong legacy effects [49], and that the intensity and duration of a single land use type can have a large effect on recovery rate [6, 48, 50].

### Effect of restoration approach on recovery completeness

The mean time since restoration started for the studies in our database was 35.5±33.1 (SD) years (median 24 years) for passively restored sites and 18.3±16.1 years (median 15 years) for actively restored sites, as most >40-year-old sites were passively restored. Nearly all logged sites (80%) were passively restored. Nonetheless, floral and faunal abundance and diversity, and biogeochemical functions recovered within a decade in most former logged sites. Seventy-nine percent of mining studies were actively restored using a combination of planting and either soil amendments or recontouring topography (Fig 2b) with the remaining sites left to regenerate passively. Most mining studies reported that the measured metrics had achieved reference levels, which suggests that the intensive efforts to restore abiotic conditions, that are often legislatively mandated, are having the intended effect in these sites. Likewise, past studies have highlighted the importance of establishing appropriate soil, topographic, and hydrologic conditions to facilitate recovery of biogeochemical functions in more degraded systems [51, 52].

**Fig 5:**
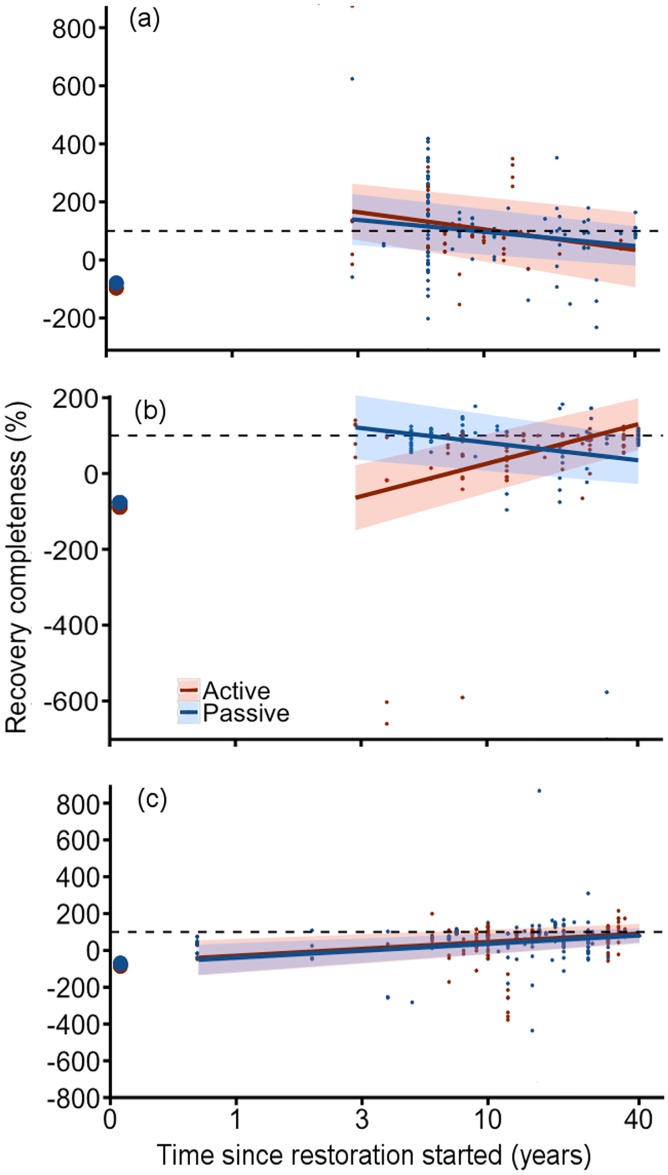
Recovery completeness in actively and passively restored former agricultural sites over time. (a) Abundance, (b) diversity, and (c) biogeochemical functions. Colored lines illustrate means and shaded areas indicate 95% confidence intervals of predicted recovery values based on models. Confidence intervals overlapping the black dashed lines indicate full recovery. Circles at time 0 indicate the mean degraded value before restoration started calculated from raw data. Small dots represent raw data. Note log scale for time since restoration on x-axis. See ‘Materials and methods‘ for modeling details.

In former agricultural land, both actively and passively restored sites showed highly variable rates of recovery, and actively restored sites did not show consistently faster or more complete recovery than passively restored sites either when comparing all studies (Fig 5; S2f–S2h and S3f–S3h Tables) or just the few studies that directly compared active and passive restoration approaches (S2i Table). Forest region and time since restoration started explained substantially more of the variation in recovery of abundance and biogeochemical functions than did restoration approach **(**S2f–S2h and S3f–S3h Tables). Restoration approach and time since restoration started had an interactive effect on diversity recovery (Fig 5b, S2g and S3g Tables). Diversity was initially lower in actively restored sites, but increased to reference levels within 10–20 years. Average diversity in passively regenerating sites overlapped with reference values regardless of time since restoration started, suggesting a progressive replacement of open field species by forest species. Similarly, previous reviews [53, 54] found almost as many studies with positive effects as with negative effects of agricultural land abandonment on plant and animal diversity.

Past studies comparing active and passive forest restoration show mixed effects of the value added of actively restoring forests. Bonner et al. [6] reported that tree planting on degraded tropical land had relatively short-lived positive effects on above-ground carbon sequestration, as compared to passive recovery. Curran et al. [9] showed positive effects of active tropical forest restoration on biodiversity, plant community similarity to reference forests, and ecosystem functions; they, however, used simulation models and selected only models explaining a significant amount of deviance that represented “the best case scenario”.

Given the large investments of money and effort in active forest restoration at a global scale, it is surprising that we did not find consistently positive effects of active as compared to passive restoration on recovery of former agricultural land, for which there are a number of possible explanations. First, we anticipated that actively restored sites would be more degraded than passively restored sites, which would confound the effects of active restoration. We found, however, that initial degradation levels in actively or passively restored sites were similar (S2b Fig). On the other hand, diversity was higher in younger passively restored sites than in actively restored ones (Fig 5b). This suggests that more resilient sites are being left to passive recovery and that early successional, ruderal species could be suppressed by active restoration treatments. Second, we analyzed data from published studies that often tested the effectiveness of different and novel restoration strategies. Some active restoration strategies are certain to influence recovery more than others making it challenging to draw conclusions about “active restoration” as a broad category. Third, specific objectives of individual restoration projects vary. For example, some projects are focused on restoring one or a few target species or a certain function (e.g., carbon sequestration) which may not accelerate the recovery of other metrics [55, 56]. Fourth, recent comparative analyses, particularly in the tropics, suggest that passive forest recovery is highly stochastic and not always predictable by stand age, making generalizations about forest recovery challenging [57]. Finally, the outcome of specific restoration projects depends not only on the restoration approach selected and biophysical conditions of the site, but is determined in large part by a complex set of socio-economic, institutional, and legal/policy drivers that are difficult to quantify [58] and were not included in our analysis due to a lack of data.

### Global patterns of forest restoration success

Patterns emerging from our synthesis of global forest restoration efforts support some general conclusions and recommendations. First, our study shows that many forests can recover floral and faunal abundance and diversity relatively quickly, an important result given the growing interest in forest restoration and the need for the associated services that these forests provide. Biogeochemical functions recover over time in most cases, which is noteworthy given the increasing focus on restoring forests to sequester carbon (e.g. REDD+ schemes) [59].

Second, our results clearly show that metrics respond variably to restoration actions [55, 56]. Nonetheless, many of the large-scale forest restoration initiatives set lofty goals to simultaneously restore ecological integrity, biodiversity, climate resilience, and a range of social goals across millions of hectares [3], despite the fact that specific ecological restoration objectives are often conflicting and variable depending on localized social and ecological constraints. Therefore, it is important that individual restoration projects clearly define measurable objectives from the outset to evaluate the efficacy of active restoration methods in achieving specific desired outcomes [60].

Third, this and many previous studies show [14, 61, 62] that some formerly forested ecosystems, particularly logged sites, recover quickly with minimal human intervention. Hence, passive recovery should be recognized as a viable and less expensive restoration option in cases where initial recovery may be rapid and the approach fits with broader project goals. Active restoration methods should be carefully tailored to the resilience of the forest being restored to effectively allocate the frequently scarce restoration resources [10]. We recommend that land managers wait a few years to observe the rate and direction of natural recovery, before investing in active restoration efforts [63].

Fourth, more studies are needed that compare outcomes of passive recovery and of multiple restoration actions in the same system at a meaningful spatial and temporal scale [6, 64]. Less than 10% of the studies we reviewed compared active and passive restoration methods in the same sites, yet comparisons of different methods in separate sites of varying ages are always plagued by confounding factors [6, 65]. Within-site comparisons, however, need not be resource intensive. For example, leaving a small area for natural regeneration in an otherwise actively restored site provides an important test of the value added of active restoration efforts. Moreover, land managers often use similar restoration methods in a given region; coordination of restoration efforts and communication and comparison of results can allow for more robust generalizations about the efficacy of specific restoration efforts. Finally, carefully planning straightforward, repeatable monitoring methods make it more feasible to collect long-term data that are critical to evaluating the efficacy of restoration methods.

## Supporting information

S1 Fig (a) Relationship between the original response ratio (RR) and transformed RR; (b) Normal quantile plot of RR raw data. (BMP)

S2 Fig Degradation level. (a) After all previous land-use types and (b) in actively and passively restored sites after agriculture only.
(BMP)

S1 File Details on database compilation and variables. (DOCX)

S2 File PRISMA 2009 flow diagram. (DOC)

S3 File PRISMA 2009 checklist. (DOC)

S1 Table Distribution of number of variables and studies in each category. (DOCX)

S2 Table Results of model comparisons. (DOCX)

S3 Table Relative importance of factors estimated by model averaging. (DOCX)
