# Supplementary figures and images for: A global review of past land use, climate, and active vs. passive restoration effects on forest recovery
Source: PLoS One. 2017 Feb 3;12(2):e0171368. doi: 10.1371/journal.pone.0171368 (PMC5291368; doi:10.1371/journal.pone.0171368)

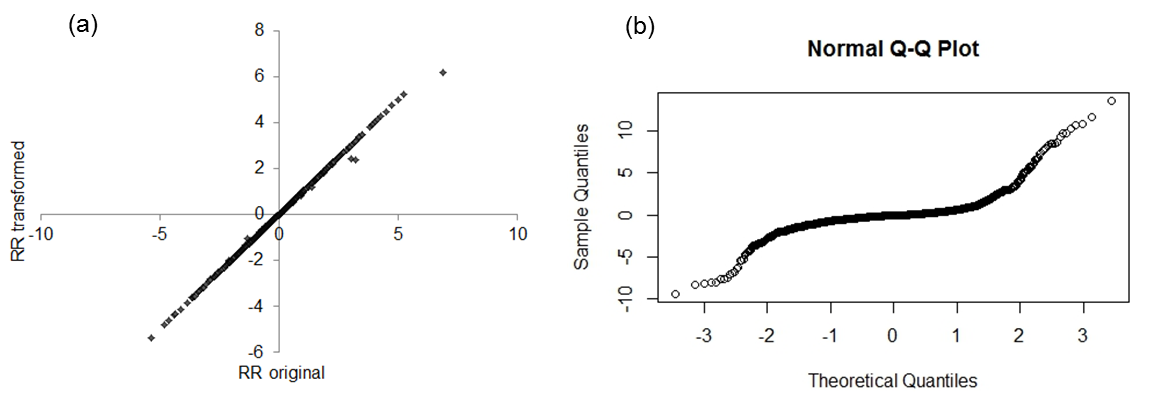

Supplement: S1 Fig — (BMP) [file pone.0171368.s001.bmp]

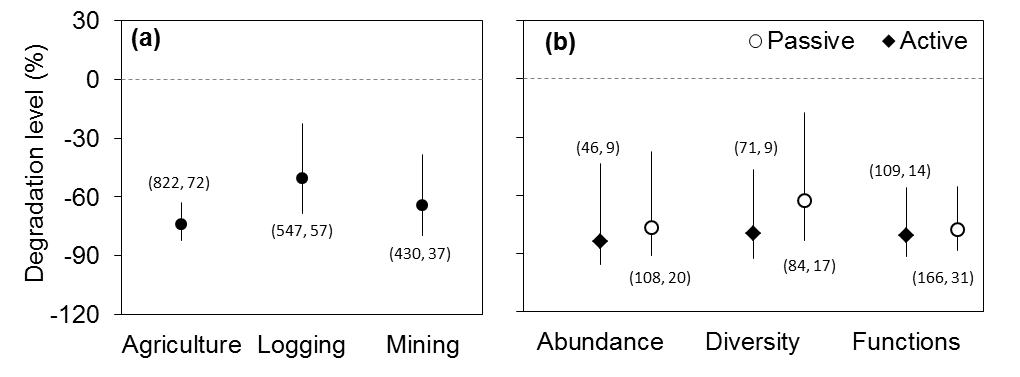

Supplement: S2 Fig — (a) After all previous land-use types and (b) in actively and passively restored sites after agriculture only. (BMP) [file pone.0171368.s002.bmp]
