# Supplementary material for: A global review of past land use, climate, and active vs. passive restoration effects on forest recovery
Source: PLoS One. 2017 Feb 3;12(2):e0171368. doi: 10.1371/journal.pone.0171368 (PMC5291368; doi:10.1371/journal.pone.0171368)
Supplement: S1 File — (DOCX) [file pone.0171368.s003.docx]

**S1 File. Details on database compilation and variables.**

*Database compilation*

As a starting point, we compiled forest studies from the databases of Rey Benayas et al. [1], Meli et al. [2], and Jones and Schmitz [3]; we found 13, 2, and 44 studies in these databases that met our specific criteria. These three previous papers focused on recovery of different ecosystem types and on restoration of biodiversity and ecosystem services generally, whereas our study focused specifically on the effects of various factors (described in detail below) on forest recovery. To update and expand this first list of studies, we conducted a systematic literature search on ISI Web of Science using the following search terms: (restorat* OR reforest* OR regenerat* OR recovery OR resilience) AND (seeding OR planting OR revegetation) AND (carbon OR biomass OR nitrogen OR organic matter) AND (forest) on 1 December 2014.

We focused on papers evaluating forest recovery after three major past land-use types, namely agriculture, logging, and mining. We did not include studies of recovery after natural disturbances such as hurricanes or fires, or afforestation and commercial forestry studies as they do not fall within the definition of ecological restoration that we used [[13](#_ENREF_13)]. Afforestation and commercial forestry include planting trees, often non-native species, into historically non-forested habitats or solely for commercial timber production, respectively. We focused on terrestrial forests and did not include shrublands, swamps, mangroves, or greenhouse experiments.

We included 107 additional studies from this search (65% of total database), resulting in a final database with 166 studies and 1,804 response variables. Thus, the majority of this database compiled for this study is new and based on a literature search conducted specifically for this study, rather than merely an expansion of previous databases.

*Description of database variables*

**Citation**: Reference information for the study.

**RV original**: Original response variable measured in the study.

**Latitude / Longitude**: Geographical coordinates of the study site.

**Forest region**: Tropical or temperate depending on the latitude. All forest located <23.5° in latitude were considered as tropical; all forest located in latitudes >23.5° were considered as temperate.

**Precip**: Precipitation (i.e., wet, dry) as described by authors of the original study. When not categorized by the authors, we considered wet forests to be those receiving a mean annual precipitation of >2000 mm per year without strong seasonality; all forest below this value or with an extended dry season were classified as dry.

**Land-use type**: Past land use in the study site (i.e., agriculture, logging, mining). We included only those papers referring to agriculture, logging or mining activities. Agricultural and mined sites were usually completely deforested. Logged sites were subjected to varying levels of timber extraction, in most cases selective harvesting but sometimes intensive logging or clearcutting.

**ResApp**: Restoration approach. Passive restoration when the land use ceased and the forest recovered without further human intervention, and active restoration for those sites that were actively restored (primarily by planting trees, but also by amending soil and recontouring topography).

**Time**: Time interval in years (log-transformed) between the completion of last restoration action and measurement of response variables.

**Metric**: Metric type (i.e., abundance, diversity, and biogeochemical function) measured in primary studies. In these studies, abundance measures included flora and fauna biomass and counts of individuals and cover. Diversity measures included species, genus or family richness, and evenness. We found few cases of community similarity indices. We categorized native and non-native species separately. Ecosystem functions included measurements of biogeochemical processes, specifically concentrations and fluxes of carbon, nitrogen, and phosphorus. We distinguished between above- and below-ground data for carbon. Nitrogen and phosphorus were primarily from below-ground, so data were not separated.

**EcoFx**: Specific biogeochemical function measured (i.e., above-ground carbon, below-ground carbon, nitrogen, and phosphorus; see previous variable).

**Life form**: Invertebrates, vertebrates, and vegetation. We did not have sufficient data to compare a more exhaustive list of life forms (e.g. comparing tree and herbs, or mammals, birds and reptiles).

**Reference**: Measure in the reference forest.

**Degraded**: Measure in the degraded forest.

**Restored**: Measure in the restored forest.

**Effect**: Sign of the response ratio. See main text for more details.

**RRcom**: Response ratio calculated as the ln ratio of each measure in the recovered/restored relative to reference forest (recovery completeness). See main text for additional details.

**RRdeg**: Response ratio calculated as the ln ratio of each measure in degraded relative to reference forest (degradation level). See main text for additional details.

**References**

1. Rey Benayas JM, Newton AC, Diaz A, Bullock JM. Enhancement of biodiversity and ecosystem services by ecological restoration: a meta-analysis. Science. 2009;325:1121-4.

2. Meli P, Benayas JMR, Balvanera P, Ramos MM. Restoration enhances wetland biodiversity and ecosystem service supply, but results are context-dependent: a meta-analysis. PloS one. 2014;9(4):e93507.

3. Jones HP, Schmitz OJ. Rapid recovery of damaged ecosystems. PLoS ONE. 2009;4(5):e5653.
