# Supplementary material for: A global review of past land use, climate, and active vs. passive restoration effects on forest recovery
Source: PLoS One. 2017 Feb 3;12(2):e0171368. doi: 10.1371/journal.pone.0171368 (PMC5291368; doi:10.1371/journal.pone.0171368)
Supplement: S2 File — (DOC) [file pone.0171368.s004.doc]

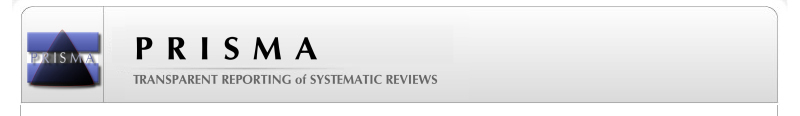
**PRISMA 2009 Flow Diagram**

**Screening**

**Included**

**Eligibility**

**Identification**

Records identified through database searching
(n = 1,279)

Additional records identified through other sources
(n = 59) (from previous databases)

Records after duplicates removed
(n = 1,324)

Records screened
(n = 1,324)

Records excluded
(n = 818)

Full-text articles assessed for eligibility
(n = 506)

Full-text articles excluded, with reasons
(n = 340)

Studies included in qualitative synthesis
(n = 166)

Studies included in quantitative synthesis (meta-analysis)
(n = 166)
