# Supplementary material for: A global review of past land use, climate, and active vs. passive restoration effects on forest recovery
Source: PLoS One. 2017 Feb 3;12(2):e0171368. doi: 10.1371/journal.pone.0171368 (PMC5291368; doi:10.1371/journal.pone.0171368)
Supplement: S1 Table — (DOCX) [file pone.0171368.s006.docx]

**S1 Table. Distribution of number of response variables (i.e. data points) and studies in each category.**

|  |  | Number | |
| --- | --- | --- | --- |
|  | Category | Variables | Studies |
| *Metric type* | Abundance | 679 | 93 |
|  | Diversity | 405 | 82 |
|  | Functions | 720 | 91 |
| *Life form (abundance)* | Invertebrates | 180 | 28 |
|  | Vegetation | 423 | 58 |
|  | Vertebrates | 76 | 14 |
| *Life form (diversity)* | Invertebrates | 159 | 32 |
|  | Vegetation | 185 | 41 |
|  | Vertebrates | 61 | 18 |
| *Ecosystem function* | Aboveground carbon | 218 | 45 |
|  | Belowground carbon | 275 | 59 |
|  | Nitrogen | 161 | 38 |
|  | Phosphorus | 66 | 27 |
| *Forest region* | Temperate | 770 | 73 |
|  | Tropical | 1034 | 95 |
| *Precipitation* | Wet | 1055 | 92 |
|  | Dry | 749 | 76 |
| *Land-use type* | Agriculture | 822 | 74 |
|  | Logging | 551 | 57 |
|  | Mining | 431 | 37 |
| *Restoration approach* | Passive | 1106 | 112 |
|  | Active | 698 | 66 |
