# Supplementary material for: A global review of past land use, climate, and active vs. passive restoration effects on forest recovery
Source: PLoS One. 2017 Feb 3;12(2):e0171368. doi: 10.1371/journal.pone.0171368 (PMC5291368; doi:10.1371/journal.pone.0171368)
Supplement: S2 Table — (DOCX) [file pone.0171368.s007.docx]

**S2 Table. Results of model comparisons.**

Null model used was ln Response Ratio ~ 1. For each model, we report k (the number of parameters in the model including the intercept and the residual error estimates), the log-likelihood log(L), Akaike information criterion (AIC), ΔAIC = AIC_i_-minAIC, and Akaike weight (w_i_). Models are ordered in terms of ΔAIC.

|  | Model | *k* | log(L) | AIC | ∆AIC | w_i_ |
| --- | --- | --- | --- | --- | --- | --- |
| *a) Degradation level* | |  |  |  |  |  |
|  | Land-use type | 5 | -4249.70 | 8509.40 | 0 | 0.27 |
|  | Null model | 3 | -4252.00 | 8510.10 | 0.65 | 0.19 |
|  | Forest region | 4 | -4251.70 | 8511.30 | 1.86 | 0.11 |
|  | Land-use type + Forest region | 6 | -4249.70 | 8511.30 | 1.91 | 0.10 |
|  | Land-use type + Precipitation | 6 | -4249.70 | 8511.40 | 1.97 | 0.10 |
|  | Land-use type * Precipitation | 8 | -4248.00 | 8512.00 | 2.56 | 0.07 |
|  | Precipitation | 4 | -4252.04 | 8512.07 | 2.64 | 0.07 |
|  | Forest region + Precipitation | 5 | -4251.65 | 8513.29 | 3.86 | 0.04 |
|  | Forest region * Precipitation | 6 | -4251.02 | 8514.04 | 4.61 | 0.03 |
|  | Land-use type * Forest region | 8 | -4249.32 | 8514.63 | 5.20 | 0.02 |
| *b) Overall recovery* | |  |  |  |  |  |
|  | Metric type * Time | 8 | -3382.96 | 6781.92 | 0.00 | 0.43 |
|  | Metric type + Time | 6 | -3385.24 | 6782.47 | 0.55 | 0.33 |
|  | Metric type + Land-use type | 7 | -3385.29 | 6784.59 | 2.67 | 0.11 |
|  | Metric type + Forest region | 6 | -3386.83 | 6785.66 | 3.75 | 0.07 |
|  | Metric type * Forest region | 8 | -3385.47 | 6786.94 | 5.02 | 0.03 |
|  | Metric type | 5 | -3389.52 | 6789.05 | 7.13 | 0.01 |
|  | Metric type * Land-use type | 11 | -3383.74 | 6789.49 | 7.57 | 0.01 |
|  | Metric type + Time | 6 | -3389.51 | 6791.02 | 9.11 | 0 |
|  | Metric type * Time | 8 | -3388.52 | 6793.04 | 11.12 | 0 |
|  | Land-use type + Time | 6 | -3397.43 | 6806.86 | 24.95 | 0 |
|  | Land-use type * Time | 8 | -3396.93 | 6809.86 | 27.94 | 0 |
|  | Forest region + Time | 5 | -3402.00 | 6814.00 | 32.08 | 0 |
|  | Time | 4 | -3403.74 | 6815.48 | 33.57 | 0 |
|  | Forest region * Time | 6 | -3401.93 | 6815.86 | 33.94 | 0 |
|  | Land-use type | 5 | -3403.27 | 6816.55 | 34.63 | 0 |
|  | Precipitation + Time | 5 | -3403.74 | 6817.48 | 35.56 | 0 |
|  | Land-use type + Forest region | 6 | -3402.99 | 6817.98 | 36.07 | 0 |
|  | Land-use type + Precipitation | 6 | -3403.06 | 6818.13 | 36.21 | 0 |
|  | Precipitation * Time | 6 | -3403.72 | 6819.43 | 37.52 | 0 |
|  | Forest region | 4 | -3406.07 | 6820.14 | 38.22 | 0 |
|  | Land-use type * Precipitation | 8 | -3402.29 | 6820.57 | 38.66 | 0 |
|  | Land-use type * Forest region | 8 | -3402.84 | 6821.68 | 39.76 | 0 |
|  | Forest region + Precipitation | 5 | -3405.97 | 6821.95 | 40.03 | 0 |
|  | Null model | 3 | -3407.99 | 6821.98 | 40.07 | 0 |
|  | Forest region * Precipitation | 6 | -3405.89 | 6823.77 | 41.86 | 0 |
|  | Precipitation | 4 | -3407.98 | 6823.96 | 42.04 | 0 |
| *c) Species abundance* | |  |  |  |  |  |
|  | Time | 4 | -1473.02 | 2954.04 | 0 | 0.15 |
|  | Land-use type + Time | 6 | -1471.43 | 2954.85 | 0.81 | 0.10 |
|  | Land-use type + Precipitation + Time | 7 | -1470.58 | 2955.16 | 1.13 | 0.08 |
|  | Null model | 3 | -1474.71 | 2955.42 | 1.38 | 0.07 |
|  | Precipitation + Time | 5 | -1472.86 | 2955.72 | 1.68 | 0.06 |
|  | Forest region + Time | 5 | -1472.90 | 2955.79 | 1.75 | 0.06 |
|  | Land-use type * Precipitation + Time | 9 | -1469.29 | 2956.58 | 2.55 | 0.04 |
|  | Land-use type + Forest region + Time | 7 | -1471.37 | 2956.75 | 2.71 | 0.04 |
|  | Precipitation | 4 | -1474.52 | 2957.04 | 3.01 | 0.03 |
|  | Life Form + Time | 8 | -1470.54 | 2957.08 | 3.04 | 0.03 |
|  | Land-use type + Precipitation * Time | 4 | -1474.54 | 2957.09 | 3.05 | 0.03 |
|  | Forest region | 6 | -1472.55 | 2957.09 | 3.06 | 0.03 |
|  | Forest region + Precipitation + Time | 6 | -1472.68 | 2957.36 | 3.32 | 0.03 |
|  | Life Form * Time | 8 | -1470.73 | 2957.45 | 3.42 | 0.03 |
|  | Forest region * Time | 6 | -1472.74 | 2957.48 | 3.44 | 0.03 |
|  | Precipitation * Time | 6 | -1472.75 | 2957.5 | 3.47 | 0.03 |
|  | Land-use type | 5 | -1473.83 | 2957.66 | 3.62 | 0.02 |
|  | Forest region * Precipitation + Time | 7 | -1472.12 | 2958.24 | 4.21 | 0.02 |
|  | Life Form | 5 | -1474.19 | 2958.38 | 4.34 | 0.02 |
|  | Land-use type + Precipitation | 6 | -1473.25 | 2958.5 | 4.46 | 0.02 |
|  | Forest region + Precipitation | 5 | -1474.29 | 2958.59 | 4.55 | 0.01 |
|  | Land-use type * Time | 8 | -1471.30 | 2958.6 | 4.57 | 0.01 |
|  | Land-use type + Forest region * Time | 8 | -1471.33 | 2958.66 | 4.62 | 0.01 |
|  | Land-use type * Forest region + Time | 9 | -1470.53 | 2959.05 | 5.02 | 0.01 |
|  | Forest region + Precipitation * Time | 7 | -1472.61 | 2959.22 | 5.18 | 0.01 |
|  | Land-use type + Forest region | 6 | -1473.83 | 2959.66 | 5.62 | 0.01 |
|  | Forest region * Precipitation | 6 | -1474.02 | 2960.05 | 6.01 | 0.01 |
|  | Land-use type * Precipitation | 8 | -1472.09 | 2960.18 | 6.14 | 0.01 |
|  | Land-use type * Forest region | 8 | -1473.13 | 2962.27 | 8.23 | 0 |
| *d) Species diversity* | |  |  |  |  |  |
|  | Forest region * Time | 6 | -613.17 | 1238.35 | 0 | 0.14 |
|  | Forest region + Precipitation * Time | 7 | -612.20 | 1238.40 | 0.05 | 0.13 |
|  | Forest region | 4 | -615.57 | 1239.14 | 0.79 | 0.09 |
|  | Forest region + Time | 5 | -614.71 | 1239.42 | 1.08 | 0.08 |
|  | Life Form * Time | 8 | -611.76 | 1239.53 | 1.18 | 0.07 |
|  | Precipitation * Time | 6 | -614.15 | 1240.29 | 1.95 | 0.05 |
|  | Null model | 8 | -612.38 | 1240.75 | 2.40 | 0.04 |
|  | Land-use type + Precipitation * Time | 3 | -617.38 | 1240.75 | 2.41 | 0.04 |
|  | Forest region + Precipitation | 5 | -615.48 | 1240.97 | 2.62 | 0.04 |
|  | Time | 4 | -616.49 | 1240.99 | 2.64 | 0.04 |
|  | Forest region + Precipitation + Time | 6 | -614.55 | 1241.09 | 2.75 | 0.03 |
|  | Land-use type + Forest region * Time | 8 | -612.91 | 1241.82 | 3.47 | 0.02 |
|  | Land-use type | 5 | -616.00 | 1242.00 | 3.66 | 0.02 |
|  | Land-use type + Forest region | 6 | -615.14 | 1242.28 | 3.93 | 0.02 |
|  | Land-use type + Time | 6 | -615.21 | 1242.42 | 4.08 | 0.02 |
|  | Precipitation | 4 | -617.27 | 1242.54 | 4.19 | 0.02 |
|  | Precipitation + Time | 5 | -616.30 | 1242.60 | 4.25 | 0.02 |
|  | Forest region * Precipitation | 6 | -615.32 | 1242.63 | 4.29 | 0.02 |
|  | Land-use type + Forest region + Time | 7 | -614.33 | 1242.66 | 4.31 | 0.02 |
|  | Forest region * Precipitation + Time | 7 | -614.36 | 1242.71 | 4.37 | 0.02 |
|  | Life Form + Time | 6 | -615.45 | 1242.89 | 4.55 | 0.01 |
|  | Life Form | 5 | -616.46 | 1242.91 | 4.56 | 0.01 |
|  | Land-use type * Time | 8 | -613.77 | 1243.54 | 5.19 | 0.01 |
|  | Land-use type * Precipitation + Time | 9 | -612.78 | 1243.57 | 5.22 | 0.01 |
|  | Land-use type * Forest region | 6 | -615.89 | 1243.78 | 5.43 | 0.01 |
|  | Land-use type + Precipitation | 8 | -613.89 | 1243.78 | 5.43 | 0.01 |
|  | Land-use type * Precipitation | 8 | -613.94 | 1243.89 | 5.54 | 0.01 |
|  | Land-use type + Precipitation + Time | 7 | -615.06 | 1244.11 | 5.77 | 0.01 |
|  | Land-use type * Forest region + Time | 9 | -613.32 | 1244.65 | 6.30 | 0.01 |
| *e) Biogeochemical functions* | |  |  |  |  |  |
|  | Land-use type * Time | 8 | -1098.57 | 2213.14 | 0 | 0.36 |
|  | Land-use type + Time | 6 | -1101.06 | 2214.11 | 0.97 | 0.22 |
|  | Land-use type + Forest region + Time | 7 | -1100.9 | 2215.80 | 2.65 | 0.09 |
|  | Land-use type + Precipitation + Time | 7 | -1100.93 | 2215.86 | 2.72 | 0.09 |
|  | Land-use type + Forest region * Time | 8 | -1099.93 | 2215.87 | 2.72 | 0.09 |
|  | Land-use type + Precipitation * Time | 8 | -1100.46 | 2216.93 | 3.78 | 0.05 |
|  | Land-use type * Precipitation + Time | 9 | -1099.60 | 2217.20 | 4.06 | 0.05 |
|  | Land-use type * Forest region + Time | 9 | -1100.36 | 2218.72 | 5.57 | 0.02 |
|  | Forest region + Time | 5 | -1105.71 | 2221.41 | 8.27 | 0.01 |
|  | Forest region * Time | 6 | -1105.24 | 2222.49 | 9.34 | 0 |
|  | Time | 4 | -1107.27 | 2222.53 | 9.39 | 0 |
|  | Forest region + Precipitation + Time | 6 | -1105.66 | 2223.31 | 10.17 | 0 |
|  | Ecosystem function + Time | 7 | -1104.75 | 2223.49 | 10.35 | 0 |
|  | Land-use type | 5 | -1107.08 | 2224.16 | 11.02 | 0 |
|  | Precipitation + Time | 5 | -1107.27 | 2224.53 | 11.39 | 0 |
|  | Forest region + Precipitation * Time | 7 | -1105.38 | 2224.75 | 11.61 | 0 |
|  | Forest region * Precipitation + Time | 7 | -1105.64 | 2225.28 | 12.13 | 0 |
|  | Land-use type + Forest region | 6 | -1106.79 | 2225.58 | 12.43 | 0 |
|  | Land-use type + Precipitation | 6 | -1106.79 | 2225.59 | 12.44 | 0 |
|  | Precipitation * Time | 6 | -1107.09 | 2226.18 | 13.04 | 0 |
|  | Land-use type * Precipitation | 8 | -1105.33 | 2226.65 | 13.51 | 0 |
|  | Forest region | 4 | -1109.70 | 2227.39 | 14.25 | 0 |
|  | Ecosystem function * Time | 10 | -1103.96 | 2227.93 | 14.78 | 0 |
|  | Null model | 3 | -1111.27 | 2228.54 | 15.39 | 0 |
|  | Forest region + Precipitation | 5 | -1109.50 | 2229.01 | 15.86 | 0 |
|  | Land-use type * Forest region | 8 | -1106.57 | 2229.14 | 16.00 | 0 |
|  | Ecosystem function | 6 | -1109.11 | 2230.22 | 17.07 | 0 |
|  | Precipitation | 4 | -1111.24 | 2230.47 | 17.33 | 0 |
|  | Forest region * Precipitation | 6 | -1109.50 | 2231.01 | 17.86 | 0 |
| *f) Recovery of species abundance in agricultural sites* | | | | | | |
|  | Forest region * Time | 6 | -269.99 | 551.98 | 0 | 0.24 |
|  | Forest region * Restoration approach | 6 | -270.14 | 552.28 | 0.30 | 0.20 |
|  | Time | 4 | -272.23 | 552.47 | 0.49 | 0.19 |
|  | Null model | 3 | -274.00 | 554.00 | 2.01 | 0.09 |
|  | Forest region + Time | 5 | -272.03 | 554.07 | 2.09 | 0.08 |
|  | Restoration approach + Time | 5 | -272.20 | 554.40 | 2.42 | 0.07 |
|  | Restoration approach | 4 | -273.57 | 555.14 | 3.16 | 0.05 |
|  | Forest region | 4 | -273.89 | 555.78 | 3.80 | 0.04 |
|  | Restoration approach * Time | 6 | -272.14 | 556.29 | 4.31 | 0.03 |
|  | Forest region + Restoration approach | 5 | -273.53 | 557.05 | 5.07 | 0.02 |
| *g) Recovery of species diversity in agricultural sites* | | | | | | |
|  | Restoration approach * Time | 6 | -255.97 | 523.95 | 0 | 0.60 |
|  | Null model | 3 | -260.93 | 527.86 | 3.91 | 0.09 |
|  | Time | 4 | -260.23 | 528.45 | 4.50 | 0.06 |
|  | Forest region | 4 | -260.34 | 528.68 | 4.73 | 0.06 |
|  | Forest region + Time | 5 | -259.68 | 529.35 | 5.41 | 0.04 |
|  | Restoration approach | 4 | -260.73 | 529.45 | 5.51 | 0.04 |
|  | Restoration approach + Forest region | 5 | -259.77 | 529.54 | 5.59 | 0.04 |
|  | Restoration approach + Time | 5 | -259.90 | 529.81 | 5.86 | 0.03 |
|  | Forest region * Time | 6 | -259.03 | 530.06 | 6.11 | 0.03 |
|  | Restoration approach * Forest region | 6 | -259.55 | 531.11 | 7.16 | 0.02 |
| *h) Recovery of biogeochemical functions in agricultural sites* | | | | | | |
|  | Time | 4 | -415.65 | 839.3 | 0 | 0.30 |
|  | Forest region * Time | 6 | -413.66 | 839.32 | 0.02 | 0.30 |
|  | Forest region + Time | 5 | -415.26 | 840.51 | 1.21 | 0.16 |
|  | Restoration approach + Time | 5 | -415.54 | 841.08 | 1.77 | 0.12 |
|  | Restoration approach * Time | 6 | -415.54 | 843.08 | 3.77 | 0.05 |
|  | Null model | 3 | -419.04 | 844.08 | 4.78 | 0.03 |
|  | Forest region | 4 | -418.70 | 845.39 | 6.09 | 0.01 |
|  | Restoration approach * Forest region | 6 | -416.70 | 845.39 | 6.09 | 0.01 |
|  | Restoration approach | 4 | -419.02 | 846.04 | 6.74 | 0.01 |
|  | Restoration approach + Forest region | 5 | -418.68 | 847.36 | 8.05 | 0.01 |
| *i) Recovery in studies with direct active vs. passive comparisons* | | | | | | |
|  | Null model | 3 | -310.12 | 626.23 | 0 | 0.28 |
|  | Time | 4 | -309.67 | 627.34 | 1.11 | 0.16 |
|  | Metric type | 5 | -308.68 | 627.36 | 1.12 | 0.16 |
|  | Restoration approach | 4 | -309.89 | 627.78 | 1.55 | 0.13 |
|  | Restoration approach + Time | 5 | -309.44 | 628.88 | 2.65 | 0.07 |
|  | Metric type + Time | 6 | -308.45 | 628.89 | 2.66 | 0.07 |
|  | Metric type + Restoration approach | 6 | -308.48 | 628.96 | 2.73 | 0.07 |
|  | Restoration approach * Time | 6 | -309.12 | 630.24 | 4.01 | 0.04 |
|  | Metric type * Time | 8 | -308.01 | 632.02 | 5.78 | 0.02 |
|  | Metric type * Restoration approach | 8 | -308.32 | 632.65 | 6.41 | 0.01 |
