# Supplementary material for: A global review of past land use, climate, and active vs. passive restoration effects on forest recovery
Source: PLoS One. 2017 Feb 3;12(2):e0171368. doi: 10.1371/journal.pone.0171368 (PMC5291368; doi:10.1371/journal.pone.0171368)
Supplement: S3 Table — (DOCX) [file pone.0171368.s008.docx]

**S3 Table. Relative importance** (**Ʃ_w_)** **of each factor estimated by model averaging procedure for models in S2 Table.**

|  | Predictor | Ʃ_w_ |
| --- | --- | --- |
| a) Degradation level | Land-use type | 0.56 |
|  | Precipitation | 0.31 |
|  | Forest region | 0.29 |
|  | Land-use type * Precipitation | 0.07 |
|  | Forest region * Precipitation | 0.03 |
|  | Land-use type * Forest region | 0.02 |
| b) Overall recovery | Metric type | 1.00 |
|  | Time | 0.76 |
|  | Metric type * Time | 0.43 |
|  | Land-use type | 0.12 |
|  | Forest region | 0.10 |
|  | Metric type * Forest region | 0.03 |
|  | Metric type * Land-use type | 0.01 |
|  | Metric type * Precipitation | <0.01 |
|  | Land-use type * Time | <0.01 |
|  | Land-use type | <0.01 |
|  | Forest region * Time | <0.01 |
|  | Precipitation * Time | <0.01 |
|  | Land-use type * Precipitation | <0.01 |
|  | Land-use type * Forest region | <0.01 |
|  | Forest region * Precipitation | <0.01 |
| c) Species abundance | Time | 0.77 |
|  | Land-use type | 0.39 |
|  | Precipitation | 0.38 |
|  | Forest region | 0.27 |
|  | Life Form | 0.07 |
|  | Precipitation * Time | 0.07 |
|  | Land-use type * Precipitation | 0.05 |
|  | Forest region * Time | 0.04 |
|  | Life Form * Time | 0.03 |
|  | Forest region * Precipitation | 0.03 |
|  | Land-use type * Time | 0.01 |
|  | Land-use type * Forest region | 0.01 |
| d) Species diversity | Time | 0.72 |
|  | Forest region | 0.61 |
|  | Precipitation | 0.39 |
|  | Precipitation * Time | 0.22 |
|  | Land-use type | 0.20 |
|  | Forest region * Time | 0.16 |
|  | Life Form | 0.10 |
|  | Life Form * Time | 0.07 |
|  | Forest region * Precipitation | 0.03 |
|  | Land-use type * Precipitation | 0.02 |
|  | Land-use type * Time | 0.01 |
|  | Land-use type * Forest region | 0.01 |
| e) Biogeochemical functions | Time | 1.00 |
|  | Land-use type | 0.98 |
|  | Land-use type * Time | 0.36 |
|  | Forest region | 0.22 |
|  | Precipitation | 0.20 |
|  | Forest region * Time | 0.09 |
|  | Precipitation * Time | 0.06 |
|  | Land-use type * Precipitation | 0.05 |
|  | Land-use type * Forest region | 0.02 |
|  | Biogeochemical function type | <0.01 |
|  | Biogeochemical function type * Time | <0.01 |
|  | Forest region * Precipitation | <0.01 |
| f) Species abundance in agricultural sites | Time | 0.61 |
|  | Forest region | 0.58 |
|  | Restoration approach | 0.37 |
|  | Forest region * Time | 0.24 |
|  | Forest region * Restoration approach | 0.20 |
|  | Restoration approach * Time | 0.03 |
| g) Species diversity in agricultural sites | Time | 0.77 |
|  | Restoration approach | 0.73 |
|  | Restoration approach * Time | 0.60 |
|  | Forest region | 0.18 |
|  | Forest region * Time | 0.03 |
|  | Forest region * Restoration approach | 0.02 |
| h) Biogeochemical functions in agricultural sites | Time | 0.93 |
|  | Forest region | 0.49 |
|  | Forest region * Time | 0.30 |
|  | Restoration approach | 0.20 |
|  | Restoration approach * Time | 0.05 |
|  | Forest region * Restoration approach | 0.01 |
| 1. Direct active vs. passive comparison studies only | Time | 0.36 |
|  | Metric type | 0.33 |
|  | Restoration approach | 0.32 |
|  | Restoration approach * Time | 0.04 |
|  | Metric type * Time | 0.02 |
|  | Metric type * Restoration approach | 0.01 |
